# Genetic Diversity of Brazilian *Aedes aegypti*: Patterns following an Eradication Program

**DOI:** 10.1371/journal.pntd.0003167

**Published:** 2014-09-18

**Authors:** Fernando A. Monteiro, Renata Shama, Ademir J. Martins, Andrea Gloria-Soria, Julia E. Brown, Jeffrey R. Powell

**Affiliations:** 1 Laboratorio de Epidemiologia e Sistematica Molecular, Instituto Oswald Cruz—Fiocruz, Rio de Janeiro, Brazil; 2 Laboratório de Biologia Computacional e Sistemas, IOC – Fiocruz, Rio de Janeiro, Brazil; 3 Laboratório de Fisiologia e Controle de Artrópodes Vetores, IOC – Fiocruz, Rio de Janeiro, Brazil; 4 Department of Ecology and Evolutionary Biology, Yale University, New Haven, Connecticut, United States of America; Makerere University, Uganda

## Abstract

**Background:**

*Aedes aegypti* is the most important vector of dengue fever in Brazil, where severe epidemics have recently taken place. *Ae. aegypti* in Brazil was the subject of an intense eradication program in the 1940s and 50s to control yellow fever. Brazil was the largest country declared free of this mosquito by the Pan-American Health Organization in 1958. Soon after relaxation of this program, *Ae. aegypti* reappeared in this country, and by the early 1980s dengue fever had been reported. The aim of this study is to analyze the present-day genetic patterns of *Ae. aegypti* populations in Brazil.

**Methodology/Principal Findings:**

We studied the genetic variation in samples of 11 widely spread populations of *Ae. aegypti* in Brazil based on 12 well-established microsatellite loci. Our principal finding is that present-day Brazilian *Ae. aegypti* populations form two distinct groups, one in the northwest and one in the southeast of the country. These two groups have genetic affinities to northern South American countries and the Caribbean, respectively. This is consistent with what has been reported for other genetic markers such as mitochondrial DNA and allele frequencies at the insecticide resistance gene, *kdr*.

**Conclusions/Significance:**

We conclude that the genetic patterns in present day populations of *Ae. aegypti* in Brazil are more consistent with a complete eradication of the species in the recent past followed by re-colonization, rather than the alternative possibility of expansion from residual pockets of refugia. At least two colonizations are likely to have taken place, one from northern South American countries (e.g., Venezuela) that founded the northwestern group, and one from the Caribbean that founded the southeastern group. The proposed source areas were never declared free of *Ae. aegypti*.

## Author Summary

The mosquito, *Aedes aegypti*, was historically very important as the major vector of yellow fever, whereas today it is most notorious for being the major transmitter of dengue fever. In the 1940s and 50s, the Pan-American Health Organization organized a campaign to eradicate *Ae. aegypti* from the New World. They were partly successful, with Brazil being the largest country to be declared free of *Ae. aegypti*. Within ten years of relaxation of control efforts, *Ae. aegypti* reappeared in Brazil and today is the vector of the most intense dengue epidemics in the New World. Here, we present population genetic data that are most consistent with the species having truly been eradicated from Brazil rather than simply pushed into small refugia as a consequence of the eradication campaign. The re-infestation most likely resulted from two sources: 1) from northern S. American countries like Venezuela into northwest Brazil and 2) from the Caribbean into the southeast of the country.

## Introduction

Dengue fever is a viral disease transmitted by *Aedes* mosquitoes that occur in tropical and subtropical areas around the world. Due to a widespread distribution, this disease could be more important than malaria in terms of economic impact and morbidity [1]–[4]. It is estimated that more than two billion people (over 40% of the world's population) are at risk of infection by one or more dengue serotypes [5], [6].

Brazil is especially vulnerable to dengue epidemics, with ten times more cases than other Latin American countries during recent outbreaks [6], [7]. The main vector of dengue in Brazil is the mosquito *Aedes aegypti*, which is also a vector for yellow fever and chikungunya viruses [8]. *Ae. aegypti* is a particularly adaptable invasive species that has successfully colonized most tropical and subtropical regions of the world. This is due to the vector's highly anthropophilic behavior and ability to lay its desiccation-resistant eggs in man-made water containers, widely available in most developing countries where water distribution and sanitary conditions are rudimentary. Modern transportation and commerce have greatly contributed to the passive geographical spreading of this vector and, consequently, to disease dissemination. Due to the lack of an effective vaccine, currently, dengue control programs rely almost exclusively on vector control efforts [3], [9].

Historically, neurotoxic insecticides have been the method of choice to control *Ae. aegypti* populations [10]–[12]. However, the large-scale unregulated use of insecticides, has exerted intense selective pressures on mosquito populations leading to the development of resistant strains not only in Brazil but also worldwide [13]–[18]. This undesired outcome increases the need for the creation of new vector control methods. Several emerging technologies are based on various genetic strategies (RIDL, RNAi, HEG, Wolbachia) and are either under development or are already being field-tested [19]. Regardless of the methods employed, knowledge of the genetic variability and population subdivision of mosquito populations is pivotal for the development of rational dengue control programs.

In this context, Brazil is a particularly interesting country regarding dengue epidemiology because it has gone through a well-documented vector eradication program [10], [20]. In the first half of the 20^th^ century, when dengue was not yet a public health issue, *Ae. aegypti* populations were widespread and responsible for several yellow fever epidemics, especially in the northeast of Brazil. Motivated by the success achieved by the *Anopheles gambiae* control program, the Brazilian government launched, in 1947, an initiative to eradicate *Ae. aegypti* populations based on the use of DDT. In 1958, during the XV Conferencia Sanitária Panamericana in Puerto Rico, Brazil was declared free of *Ae. aegypti*. The species was again recorded in the late 1970's, probably as a consequence of a reduction in the efficacy of the vector control measures employed [10], [20]. The first well-documented outbreak of dengue fever in the country occurred in 1982, in Roraima state, north Brazil [21]. Today, the entire country is endemic for dengue and the last outbreak in 2013 accounted for more than 1.5 million cases (BRAZIL/Health Ministry, 2014). Therefore, dengue fever has become a major public health issue, especially because all four DENV serotypes co-circulate in the country [7].

The first studies to assess the genetic structure of Brazilian *Ae. aegypti* populations were based upon RAPD markers and revealed high levels of interpopulation genetic differentiation [22], [23]. Allozyme-based studies also indicated a high degree of genetic structure and limited gene flow between regions connected by highways and railroads, suggesting that passive mosquito dispersal is not extensive [24], [25]. Within cities, such as the densely populated Rio de Janeiro, local genetic differentiation has also been found indicating that this species has extremely limited dispersal capability [26], [27].

The analyses of mtDNA sequence data (ND4 and COI) of several Brazilian populations revealed the co-occurrence of two distinct lineages in the country [28], [29]. A study of frequencies of the *kdr* (knock-down resistance) mutations, which confer pyrethroid resistance, found at least three distinct genetic groups in 30 Brazilian populations. [18].

Microsatellites are assumed neutral, highly variable codominant markers commonly used in population genetics. However, they have never been used in a nationwide study of *Ae. aegypti* in Brazil. Here, we present the results of the analysis of 12 microsatellite loci in Brazilian *Ae. aegypti* populations in an effort to better understand the genetic structure of this vector in the country, which may lend insights into the presumed recolonization following eradication events.

## Methods

### Sample collection

*Ae. aegypti* samples were field-collected from 11 sites in Brazil (Table 1). Eggs were collected in multiple ovitraps per locality (to avoid sampling of siblings) and reared to adults for proper taxonomic identification. Samples from generation F_0_ up to F_2_ were preserved in 70–100% ethanol or dry at −80°C for further analysis. Eight previously studied populations from different countries across South, Central and North America [30] were included in the analyses (Table 1).

**Table 1:** Population information for the *Aedes aegypti* samples analyzed.

Population	Region	Gen. in the lab	N*	Year collected	Latitude	Longitude
Aracajú, Sergipe, Brazil	South America	0	24	2010	−10.909151°	−37.074454°
Goiania, Goiás, Brazil	South America	0	24	2009	−16.677715°	−49.267630°
Maceió, Alagoas, Brazil	South America	1	24	2009	−9.666252°	−35.735098°
Mossoró, Rio Grande do Norte, Brazil	South America	1	22	2009	−5.188036°	−37.344134°
Pau dos Ferros, Rio Grande do Norte, Brazil	South America	1	16	2009	−6.102490°	−38.209222°
Tucuruí, Pará, Brazil	South America	0	19	2010	−3.769528°	−49.674109°
Marabá, Pará, Brazil	South America	0	48	2011	−5.369968°	−49.116927°
Natal, Rio Grande do Norte, Brazil	South America	0	47	2010	−5.794478°	−35.210955°
São Gonçalo, Rio de Janeiro, Brazil	South America	2	20	2009	−22.827099°	−43.054379°
Cachoeiro do Itapemerim, Espirito Santo, Brazil	South America	2	23	2008	−20.849368°	−41.113221°
Cachoeiro do Itapemerim, Espirito Santo, Brazil	South America	1/2	43	2012/2013	−20.849368°	−41.113221°
Jacobina, Bahia, Brazil	South America	**0**	92	2013	−11.185452°	−40.536079°
Bolívar, Venezuela	South America	2	48	2004	6.358480°	−63.580581°
Zulia, Venezuela	South America	2	47	2004	9.967492°	−72.520483°
Houston, Texas, USA	North America	0	29	2009	29.760196°	−95.369396°
Coatzacoalcos, Mexico	Central America	0	50	2008	18.149988°	−94.433299°
Pijijiapan, Mexico	Central America	1	47	2008	15.685171°	−93.212254°
Dominica	Caribean	0	48	2009	15.414999°	−61.370976°
Puerto Rico	Caribean	0	47	2011	18.220833°	−66.590149°
Miami, Florida, USA	North America	0	47	2010	25.788969°	−80.226439°

*Number of individuals collected.

### DNA extraction and amplification

Total genomic DNA was extracted with the DNeasy Kit (Qiagen) following the manufacturer's protocol. Individual genotypes were scored for 12 previously published microsatellite loci [30], [31].

### Data analysis

Microsatellite alleles were scored using Gene Mapper software (Applied Biosystems). The experiments were performed in the Yale Laboratory using the same ABI machine as used by Brown *et al.* [30] and alleles scored in accordance with that publication, so the data presented here are directly comparable to data in Brown *et al.* [30].

To infer the statistical reliability of our markers, each locus was tested for deviations from Hardy-Weinberg expectations on the web version of Genepop v1.2 [32], [33]. The same program was used to test all loci pairs for linkage disequilibrium (LD). Markov chain parameters were set at 10,000 dememorizations, 1,000 batches and 10,000 iterations per batch for both HWE and LD. Critical significance levels were corrected for multiple tests using the Bonferroni correction. The probability of null allele occurrence in each locus within each population was calculated using MicroChecker v2.2.3 [34]. When null alleles were found, FreeNA [35] was used to infer the extent of bias imputed by their presence on F_ST_ values. Genetic diversity per locus and in each population was estimated by unbiased expected heterozygosity using GenALEx v6.5 [36]. The same program was used to compute allele frequencies for all loci across populations and for the Analysis of Molecular Variance (AMOVA). Sample size corrected allelic richness and percentage of private alleles were calculated using HP-Rare v1.0 [37], [38]. The software Arlequin v3.5.1.2 [39] was used to compute F_ST_ values and their significance between all pairs of populations with 1,000 permutations. Cavalli-Sforza and Edwards distances were computed using the software package Phylip 3.6 [40]. The Cavalli-Sforza distance was chosen since it has been shown to be more robust when null alleles are present [35], [41]. Programs of the Phylip package (SEQBOOT, GENEDIST, NEIGHBOR, CONSENSE) were used to construct a neighbor-joining tree with 1,000 bootstrap replicates. A factorial correspondence analysis (FCA) was performed with the software Genetix v4.0.5 [42] to better analyze the Brazilian samples. Isolation by distance was tested on the IBD web server v3.23 [43] and also through a Mantel test of correlation between geographical (LnKm) and genetic distance matrices (F_ST_/(1-F_ST_)). For both analyses significance was inferred with 1,000 permutations.

The Bayesian approach used in the software STRUCTURE v2.3.2 [44] was used to infer the number of genetic clusters (K) in the whole data set, without prior information of sampling locations. An admixture model was used where alpha was allowed to vary and independent allele frequencies were assumed with lambda set to one. We performed ten independent runs for each value of K (K = 1 to the maximum supposed number of populations) with a burn-in phase of 200,000 iterations followed by 600,000 replications. The program Structure Harvester v0.6.93 [45] was used to summarize these results and determine the most likely number of clusters by calculating ΔK [46]. Results from STRUCTURE were summarized with the program CLUMPP v1.1.2 [47] and visualized using the program Distruct v1.1 [48]. The program GeneClass2 v2.0 [49] was used for self-assignment tests to infer the degree to which an individual mosquito could be assigned to a specific population. Self-assignment tests were performed with reference populations based on geography and clusters identified by the program STRUCTURE.

## Results

### Marker assessment

Although 15 of the 1,244 (1.2%) locus-by-locus tests for LD remained significant after Bonferroni correction, no two loci were consistently correlated across populations. Eleven of the 231 (4.76%) F_IS_ values deviated significantly from Hardy-Weinberg expectations at the 5% significance level after sequential Bonferroni correction (Table S1). Of the 20 population-specific tests for each marker, zero (AC1, AC2, AC4, CT2, AG5, B2 and B3), one (AG1, AG2 and A1), three (AC5) and five (A9) tests were significant. For A9, all significant tests resulted from an excess of homozygotes, probably due to null alleles as reported in Brown *et al.* [30]. Micro-checker results suggest that locus A9 has a high probability of having null alleles in 11 populations and AC5 in five. Null allele frequency varied from 0 to 0.32 among populations for the A9 locus and 0 to 0.21 for the AC5 locus (Table S2). Other loci had null allele frequencies predicted as well (in four populations for AG2, three populations for AC1, two populations for B3 and one population for AC4, AG1 and AC2), although none with frequencies >0.14 (Table S2). Null alleles at microsatellite loci are commonly found in insects [50]–[52] and have been demonstrated to be especially common in species with large population sizes [35], which is likely the case for *Ae. aegypti* populations.

The decrease in diversity caused by null alleles can lead to an overestimation of statistics such as F_ST_ and identity values [53], especially when there is low gene flow among populations [35], [54]. Nevertheless, simulation studies have shown the bias to be small for lower F_ST_ values and almost none when assignment methods are used [54]. A comparison between FreeNA corrected and non-corrected pairwise F_ST_ values shows very small deviations in our dataset (Table 2).

**Table 2:** Population pairwise F_ST_ * values for *Ae. aegypti* populations studied.

	ARA	GO	MA	MOS	PDF	TU	MAR	NAT	SG	BOL	ZUL	HOU	COA	PIJI	DOM	PR	MIA	ES08	ES12	JAC
**ARA**	-	0.18	0.13	0.03	0.06	0.14	0.12	0.13	0.12	0.20	0.23	0.21	0.34	0.19	0.21	0.16	0.14	0.15	0.15	0.16
**GO**	0.19	-	0.18	0.19	0.13	0.15	0.13	0.13	0.11	0.20	0.19	0.25	0.34	0.22	0.23	0.12	0.13	0.17	0.08	0.17
**MA**	0.15	0.19	-	0.17	0.10	0.11	0.13	0.09	0.07	0.21	0.24	0.30	0.30	0.22	0.17	0.12	0.16	0.08	0.11	0.10
**MOS**	0.03	0.20	0.19	-	0.06	0.14	0.13	0.12	0.12	0.22	0.23	0.23	0.33	0.23	0.21	0.17	0.16	0.16	0.17	0.16
**PDF**	0.06	0.13	0.11	0.06	-	0.09	0.09	0.06	0.06	0.17	0.14	0.21	0.31	0.16	0.16	0.10	0.08	0.09	0.09	0.09
**TU**	0.15	0.15	0.10	0.15	0.09	-	0.03	0.12	0.10	0.17	0.13	0.25	0.27	0.22	0.22	0.15	0.10	0.11	0.11	0.15
**MAR**	0.13	0.13	0.14	0.14	0.10	0.03	-	0.14	0.10	0.13	0.12	0.21	0.27	0.20	0.20	0.12	0.09	0.12	0.10	0.16
**NAT**	0.13	0.12	0.09	0.12	0.06	0.11	0.14	-	0.05	0.19	0.19	0.26	0.31	0.20	0.16	0.11	0.13	0.08	0.07	0.05
**SG**	0.12	0.11	0.08	0.14	0.06	0.09	0.10	0.06	-	0.13	0.15	0.22	0.31	0.16	0.14	0.06	0.09	0.06	0.04	0.04
**BOL**	0.20	0.20	0.21	0.22	0.17	0.16	0.13	0.20	0.12	-	0.12	0.19	0.32	0.18	0.20	0.13	0.11	0.19	0.13	0.16
**ZUL**	0.24	0.19	0.24	0.24	0.15	0.13	0.12	0.19	0.15	0.12	-	0.24	0.39	0.26	0.23	0.16	0.08	0.21	0.17	0.19
**HOU**	0.22	0.26	0.32	0.23	0.22	0.25	0.21	0.26	0.23	0.19	0.24	-	0.36	0.20	0.21	0.18	0.12	0.33	0.25	0.26
**COA**	0.35	0.35	0.30	0.33	0.32	0.27	0.27	0.31	0.31	0.32	0.39	0.36	-	0.28	0.29	0.27	0.24	0.31	0.29	0.28
**PIJI**	0.19	0.22	0.22	0.23	0.17	0.21	0.20	0.21	0.16	0.18	0.27	0.21	0.28	-	0.20	0.15	0.13	0.24	0.19	0.18
**DOM**	0.22	0.24	0.19	0.22	0.18	0.23	0.21	0.17	0.16	0.21	0.24	0.21	0.29	0.21	-	0.12	0.14	0.19	0.17	0.12
**PR**	0.16	0.12	0.13	0.18	0.11	0.14	0.12	0.12	0.07	0.14	0.16	0.19	0.27	0.16	0.13	-	0.08	0.14	0.11	0.12
**MIA**	0.15	0.13	0.16	0.16	0.08	0.09	0.09	0.13	0.10	0.12	0.08	0.12	0.24	0.13	0.15	0.08	-	0.17	0.13	0.14
**ES08**	0.16	0.17	0.07	0.17	0.09	0.11	0.12	0.09	0.06	0.20	0.21	0.34	0.30	0.24	0.21	0.15	0.17	-	0.05	0.07
**ES12**	0.16	0.08	0.11	0.18	0.09	0.10	0.11	0.07	0.04	0.14	0.17	0.26	0.29	0.20	0.18	0.12	0.14	0.05	-	0.06
**JAC**	0.16	0.16	0.10	0.16	0.09	0.14	0.16	0.05	0.05	0.17	0.20	0.26	0.29	0.19	0.13	0.13	0.15	0.07	0.06	-

*Below diagonal, F_ST_ values without correction, all significantly different from zero. Above diagonal FreeNA corrected F_ST_ values.

Aracajú – ARA; Goiania – GO, Maceió – MA; Mossoró – MOS; Pau dos Ferros – PDF; Tucuruí – TU; Marabá – MAR; Natal – NAT; São Gonçalo – SG; Bolivar – BOL; Zúlia – ZUL; Houston – HOU; Coatzacoalcos – COA; Pijijiapan – PIJI; Dominica – DOM; Puerto Rico – PR; Miami – MIA; Cachoeiro 2008 – ES08; Cachoeiro 2012 – ES12; Jacobina – JAC.

### Levels of genetic variation

**Figure 1:**
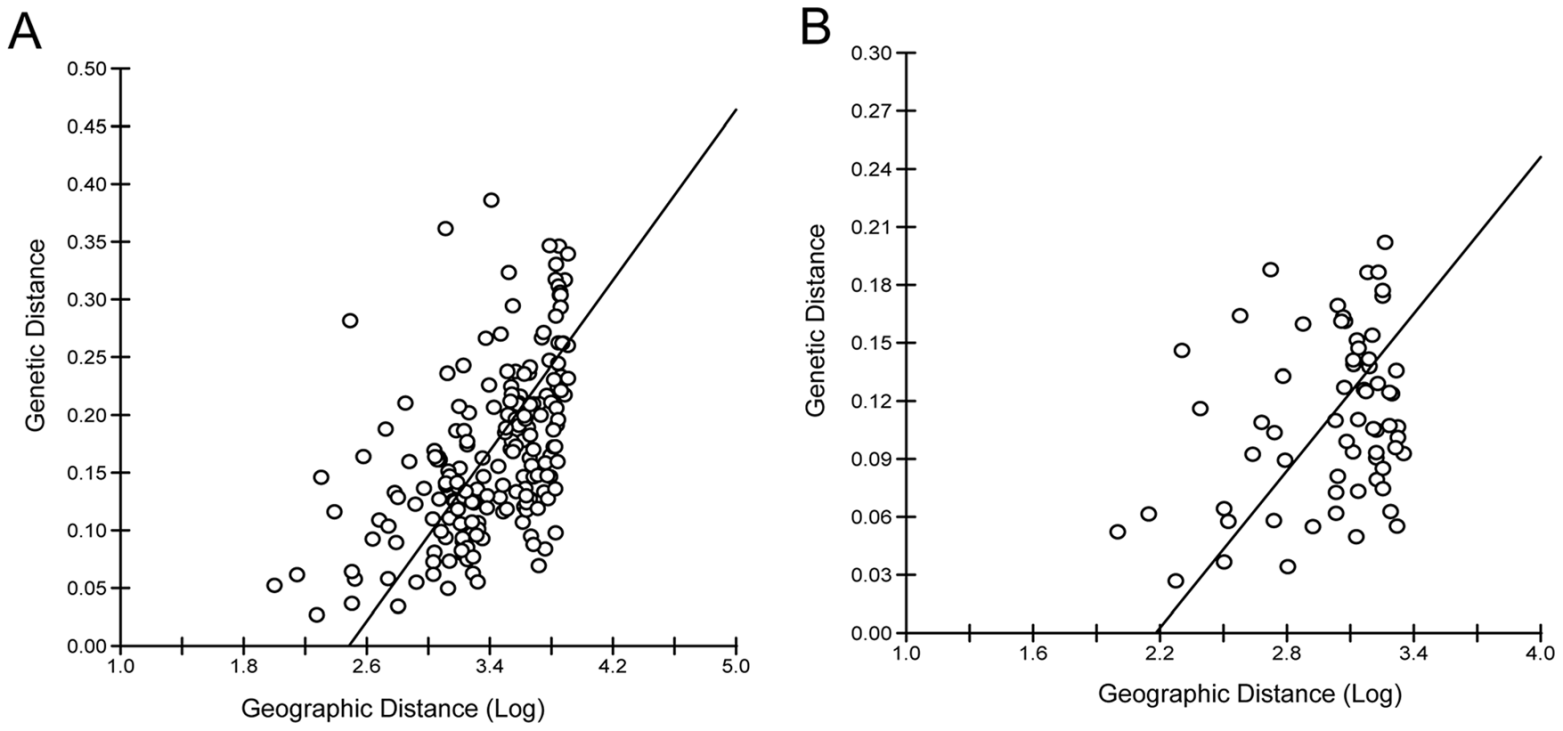
Mantel test of isolation by distance on *Ae. aegypti* populations. Scatter plots of genetic vs. geographical distances (on a logarithmic scale) for pairwise population comparisons. A- All populations (P<0.001, R^2^ = 0.53), B- only Brazilian populations (P = 0.01, R^2^ = 0.31).

Gene frequencies, heterozygosities (H_o_ and H_e_), and allelic richness for all loci studied are given in Table S3. All populations have similar diversity measures. AMOVA results show that within population differences account for 83% of the genetic variation found. Private allelic richness was low (N_p_<0.08) with only Pau dos Ferros, São Gonçalo, Dominica and Miami with estimates greater than 0.16 (Table S3). Overall F_ST_ value (F_ST_ = 0.175; 95% confidence interval 0.146–0.204) indicates a moderate level of population differentiation (Table 2). Coatzacoalcos and Houston were the only populations to have higher F_ST_ values (ranging from 0.24 to 0.38 in Coatzacoalcos and 0.11 to 0.31 in Houston). Miami and some Brazilian populations had F_ST_ values lower than 0.10 (Table 2). The genetic distance based NJ tree is reasonably consistent with geographic distances among populations (Figure S1) and was corroborated by Mantel tests of isolation by distance that found significant correlation between the geographical and genetic distance matrices (P<0.001, R^2^ = 0.53; Figure 1A). When only Brazilian samples were analyzed, weaker isolation by distance was detected by the Mantel tests (P = 0.01, R^2^ = 0.31; Figure 1B).

### Brazilian population structure and ancestry

**Figure 2:**
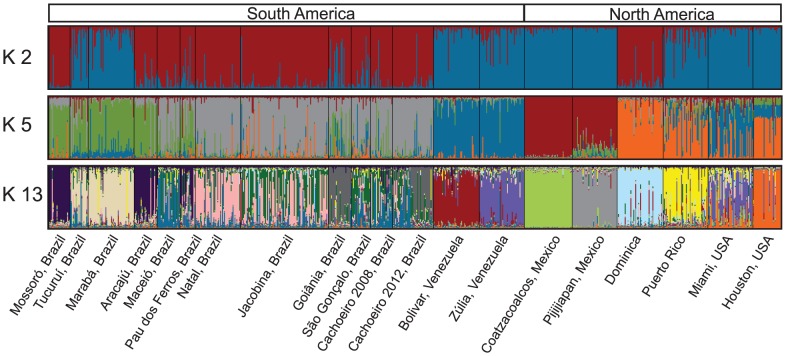
STRUCTURE bar plots for all *Ae. aegypti* populations studied. Individuals are represented by vertical bars along the plot. The height of each color represents the probability of assignment to a specific cluster. The black lines within the plots indicate population limits. Subdivision of all the individuals into K = 2 clusters, subdivision into K = 5 clusters, and into K-13 clusters. Legends below and above apply to all three K plots.

**Figure 3:**
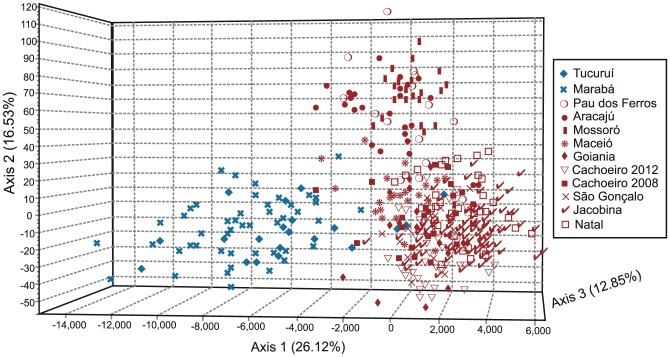
Factorial Correspondence analysis based on 12 microsatellite loci of *Ae. aegypti* populations from Brazil. Colors correspond to K = 2 cluster analysis displayed on figure 2.

A model-based clustering algorithm was used to identify subgroups with distinctive allele frequencies without prior information on population structure. In all analyses, most individuals from the same geographical origin shared similar membership coefficients in inferred clusters. The Evanno *et al.* [46] method identified K = 2 as the most likely number of clusters, but small peaks on the ΔK graph are also apparent at K = 5 and K = 13 (Figure S2). The two-cluster analysis groups include all Brazilian populations with Dominica, with the exception of Tucuruí and Marabá (Figure 2). Tucuruí and Marabá are more similar to populations from Venezuela, Mexico, Puerto Rico, and North America. Indeed, the FCA of the Brazilian samples and the NJ tree show that Tucuruí and Marabá (98% bootstrap support; Figures S1 and 3) are very different from all other Brazilian populations. In addition to these two, Mossoró, Aracajú and to some extent Pau dos Ferros also form a slightly differentiated genetic cluster on the FCA analysis (Figure 3).

While the pattern described by the above two genetic clusters is the best supported by the **Δ**K method [46], subtle substructure can be discerned by a more detailed analysis. The five-cluster STRUCTURE plot (Figure 2) shows that most populations have mixed ancestry and only Coatzacoalcos (Mexico) shows a pure genetic composition. In this analysis, the Brazilian samples from Tucuruí and Marabá now group together with Mossoró, Aracajú and, to some extent, Pau dos Ferros, consistent with the FCA analysis that indicates that these last three populations are indeed genetically differentiated as well. The thirteen-cluster plot (Figure 2) further describes the extent of *Ae. aegypti* complex genetic composition in each population. The analysis reflects admixture between groups probably due to recent gene flow among populations, although common ancestry cannot be excluded. The isolation by distance detected among samples also indicates that gene flow occurs between adjacent populations (Figure 1). The thirteen-cluster analysis further separates the Brazilian populations in five distinct clusters (Figure 2), with some mixed ancestry observed, especially in the population proximate to Rio de Janeiro (São Gonçalo), a well-known tourist destination.

Results from GeneClass2 show that when geographical locations were used as the reference populations, 83% of individuals were correctly assigned back to their population of origin. When the number of clusters inferred by STRUCTURE were used, this number increased drastically for K = 2 (94.6%) but not so much for K = 5 (92%) and even less for K = 13 (86.5%), corroborating the higher peak found for K = 2 in the Evanno plot (Figure S2).

Since STRUCTURE seems to identify the higher hierarchy in population differences [46], to better understand the relationships within the two groups identified (Blue and Red in Figure 2, K = 2 plot) we performed additional analyses. When the blue group, that encompasses Tucuruí and Marabá with EUA, Mexico, Venezuela and Puerto Rico, is analyzed; the optimal number of clusters determined by the ΔK method are K = 2 and K = 8 (Figure S3A and B). At K = 2, the two Mexican populations are differentiated from the rest and display some mixed ancestry with other populations (Figure S4). North America seems to be the most influenced by the Mexican genetic background as was already determined by Brown *et al.* [30]. Brazilian and Venezuelan populations have less background from Mexico than North America and are, therefore, similar. With K = 8, all populations except the two Brazilian ones seem to be genetically differentiated (Figure S4).

When the red group is analyzed K = 2, K = 3, and K = 5 provide some insights (Figure S5). The two-cluster analysis separates the Brazilian populations from Dominica but a high degree of mixed ancestry can be observed in Jacobina, from the Northeast of Brazil. The three-cluster analysis further differentiates the Brazilian populations showing that Mossoró, Aracajú, Pau dos Ferros and, to some extent, Natal and Maceió group together, although high levels of mixed ancestry can be observed in most populations (Figure S5). The differentiation of Mossoró, Aracajú and Pau dos Ferros from other Brazilian populations can also be seen on the FCA analysis (Figure 3). The five-cluster analysis further separates Mossoró, Aracajú and Pau dos Ferros in one cluster and shows the geographically close Maceió population to have genetic similarities with Southeastern populations (São Gonçalo and Cachoeiro). Some degree of mixed ancestry can be observed in all populations and this is most apparent in São Gonçalo, Jacobina, Maceió, Pau dos Ferros, Natal, and Cachoeiro (Figure S5). Pau dos Ferros is a small city in the state of Rio Grande do Norte that probably has both the influence of the geographically closer Mossoró and of its state capital, Natal. Interestingly, the two samples from Cachoeiro (2008 and 2012), sampled four years apart, show some degree of differentiation. In a recent study carried out in São Paulo state, Brazil, no differentiation between five sampling years was found [55].

**Figure 4:**
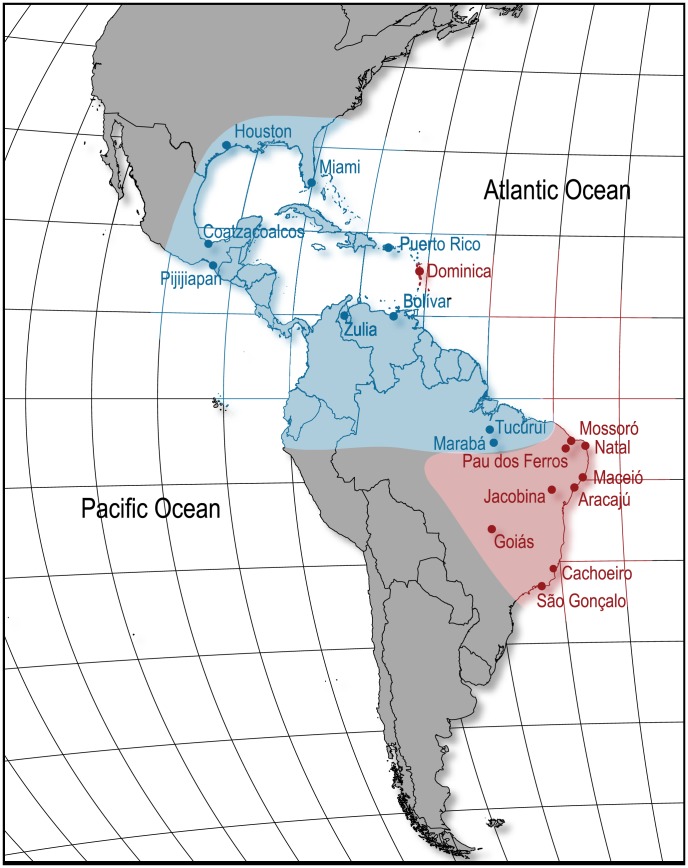
Collection sites for *Ae. aegypti* populations used in this study. Coloring indicates the two different clusters found on K = 2 STRUCTURE analysis (Figure 2).

Despite these subtle genetic patterns, we have strong evidence to conclude that Brazilian populations of *Ae. aegypti* separate into two major genetic groups with distinct affinities to populations outside Brazil as indicated in Figure 4.

## Discussion

Brazil was officially declared free of *Ae. aegypti* in 1958 [20], but reappearance of the species occurred shortly after relaxation of control measures. In its assessment of the efficacy of its eradication program, the Pan American Health Organization (PAHO) admitted that eradication had not been successful in Venezuela, Suriname, Guyana, South USA and a few Caribbean Islands [10]. It is believed that re-colonization of Brazil happened in the 1970's probably from mosquitoes from neighboring countries [7], [20].

Our results indicate that two major genetic groups are present in Brazil, one descending from Venezuela and probably other northern American countries and another one from the Caribbean (Figure 4). Bracco *et al.* [28] using the mitochondrial ND4 gene have also observed two major lineages in Brazil. The first genetic group identified suggests that mosquitoes from Venezuela and possibly the USA have contributed to the northern Brazilian population. Venezuela seems to be an important source of mosquitoes as well as dengue virus serotypes into Brazil [56]–[58]. Indeed, Silva *et al.* [59], also using the mitochondrial ND4 gene, have found that populations from the Northern states in Brazil seemed to be similar to those from Venezuela and Peru. In that study, no Caribbean Island was sampled. Venezuelan *Ae. aegypti* are highly susceptible to DENV2 virus [60] and this could be the reason Lourenço-de-Oliveira *et al.* [56] have observed that northern Brazilian populations are more susceptible to DENV2 virus than are southern ones. The second genetic group comprises Brazilian southeast and central-west populations and is genetically similar to Dominica in the Caribbean (Figure 2).

Brazil went through a nationwide vector control program based on pyrethroid insecticides from 2001 to 2009. Nevertheless, Linss *et al.* [18] detected three *kdr* genetic groups in the country (North, Northeast and Southeast-Central). Since differential selection pressures acting in the area studied could not account for their findings, the authors argued that the pattern observed could have resulted from genetic differences in the *Ae. aegypti* strains that founded those populations (Linss *et al.* [18]).

In our results, although the most important genetic break occurs between Northern populations and all others (Figures 2 and 4), the FCA also shows that Mossoró, Aracajú and, to some extent, Pau dos Ferros can be differentiated (Figure 3). When a higher cluster number is analyzed on the Bayesian clustering analysis, we see that the same three populations cluster together with the two Northern ones (Figure 2). Other studies of Brazilian *Ae. aegypti* have identified a genetic break between northern and southern populations [22]–[24], [29], [59], [61], although the exact location of the break is not always consistent. It is conceivable that the dynamics and mode of inheritance of different genetic markers can account for somewhat different patterns, e.g., cytoplasmic mtDNA *versus* nuclear genes and neutral genes *versus* selected alleles such as at insecticide resistance genes (*kdr*). The isolation by distance found within Brazilian samples suggests some connectivity among populations, so it is not surprising that the two lineages that may have initially re-invaded Brazil are now exchanging genes and perhaps merging.

The origin of these two genetic units seem reasonably clear from our data, although with only a single Caribbean sample (discounting Puerto Rico, considered part of the US) to compare, the origin of the southern lineage is less well established. Bracco *et al.* [28] suggested that Asia may have been the origin of the southern group, however, they did not sample any Caribbean Islands. Brazil has a long history of international trade within the Americas and Caribbean and only recently has this been shifted to Asian countries. Another indication that indeed Caribbean and not Asian populations might be the source of a Brazilian *Ae. aegypti* is the fact that Linss *et al.* [18] have found, in Brazilian populations, the same Caribbean *kdr* mutation allele, Val1016Ile and not Val1016Gly, that is commonly observed in Asian populations. Furthermore, Brown *et al.* [62] studying a diverse set on SNPs and nuclear gene sequence data have found that *Ae. aegypti* probably came from West Africa into the New World, where it dispersed to Asia and Australia. In their study, a Brazilian population from the Southeast (Cachoeiro) is in the same clade as Venezuelan and Caribbean populations, consistent with our findings.

While our data are consistent with the re-colonization hypothesis, we cannot exclude alternatives. The two major genetic groups observed today may have existed prior to 1958; following relaxation of vector control, the expansion from refugia within Brazil could have re-established the pattern present today. However, one expects small refugia to drift to heterogeneous gene frequencies such that subsequent expansion would lead to a mosaic of genetic units not geographically structured. Our data do not support such a scenario. Furthermore, a low genetic diversity would be expected due to a bottleneck period, which was not observed either. Measures of diversity (0.39<H_o_<0.67) and allelic richness (2.46<N_a_<4.44) are similar in Brazilian samples and other populations from the Americas, even when compared to countries where eradication did not occur () [30]. Studies with mitochondrial DNA markers (COI and ND4) have also found high genetic variability in Brazilian samples [28], [29]. Thus, while we cannot rule out incomplete eradication, for the reasons stated, recolonization from regions outside Brazil that were never declared free of *Ae. aegypti* is a simpler explanation consistent with the patterns observed in present day Brazil populations of this vector.

## Supporting Information

Figure S1 Neighbor-joining tree based on Cavalli-Sforza and Edward's chord distance computed from the allele frequencies of the 12 microsatellite loci analyzed. Unrooted tree. Numbers on branches indicate bootstrap values from 1000 replicates. Colors correspond to K = 2 cluster analysis displayed on figure 2.
(TIF)

Figure S2 Scatter plots of ΔK (A) and Log probability of the data (B) for all *Ae. aegypti* populations analyzed. ΔK plots are based on the rate of change in the log probability of the data between successive K values.
(TIF)

Figure S3 Scatter plots of ΔK (A and C) and Log probability of the data (B and D) for the two groups of *Ae. aegypti* populations. ΔK plots are based on the rate of change in the log probability of the data between successive K values. A and B – blue group from STRUCTURE analysis on figure 2 (Tucuruí, Marabá, USA, Mexico, Puerto Rico and Venezuela), C and D – red group from STRUCTURE analysis on figure 2 (Dominica and all other Brazilian populations except Tucuruí and Marabá).
(TIF)

Figure S4 STRUCTURE bar plots for *Ae. aegypti* individuals of the blue cluster group from STRUCTURE analysis on Figure 2. Vertical bars along the plot represent individuals. The height of each color represents the probability of assignment to a specific cluster. The black lines within the plots indicate population limits. Subdivision of the individuals into K = 2 clusters and subdivision into K = 8 clusters. Legends below and above are the same for the two different K plots.
(EPS)

Figure S5 STRUCTURE bar plots for *Ae. aegypti* individuals of the red cluster group from STRUCTURE analysis on Figure 2. Vertical bars along the plot represent individuals. The height of each color represents the probability of assignment to a specific cluster. The black lines within the plots indicate population limits. Subdivision of the individuals into K = 2 clusters, subdivision into K = 3 clusters and into K-5 clusters. Legends below and above apply to all three K plots.
(EPS)

Table S1 *Aedes aegypti* F_IS_ values by locus.
(DOC)

Table S2 MicroChecker v2.2.3 null allele frequency.
(DOC)

Table S3 Allele frequencies for the 12 microsatellite loci analyzed.
(DOC)
