# Correction: Genetic Diversity of Brazilian *Aedes aegypti*: Patterns following an Eradication Program

**DOI:** 10.1371/journal.pntd.0003460

**Published:** 2014-12-19

**Authors:** The PLOS Neglected Tropical Diseases Staff

The second author’s name is spelled incorrectly. The correct name is Renata Schama. The correct citation is: Monteiro FA, Schama R, Martins AJ, Gloria-Soria A, Brown JE, et al. (2014) Genetic Diversity of Brazilian Aedes aegypti: Patterns following an Eradication Program. PLoS Negl Trop Dis 8(9): e3167. doi:10.1371/journal.pntd.0003167
