# Supplementary figures and images for: Genetic Diversity of Brazilian Aedes aegypti: Patterns following an Eradication Program
Source: PLoS Negl Trop Dis. 2014 Sep 18;8(9):e3167. doi: 10.1371/journal.pntd.0003167 (PMC4169244; doi:10.1371/journal.pntd.0003167)

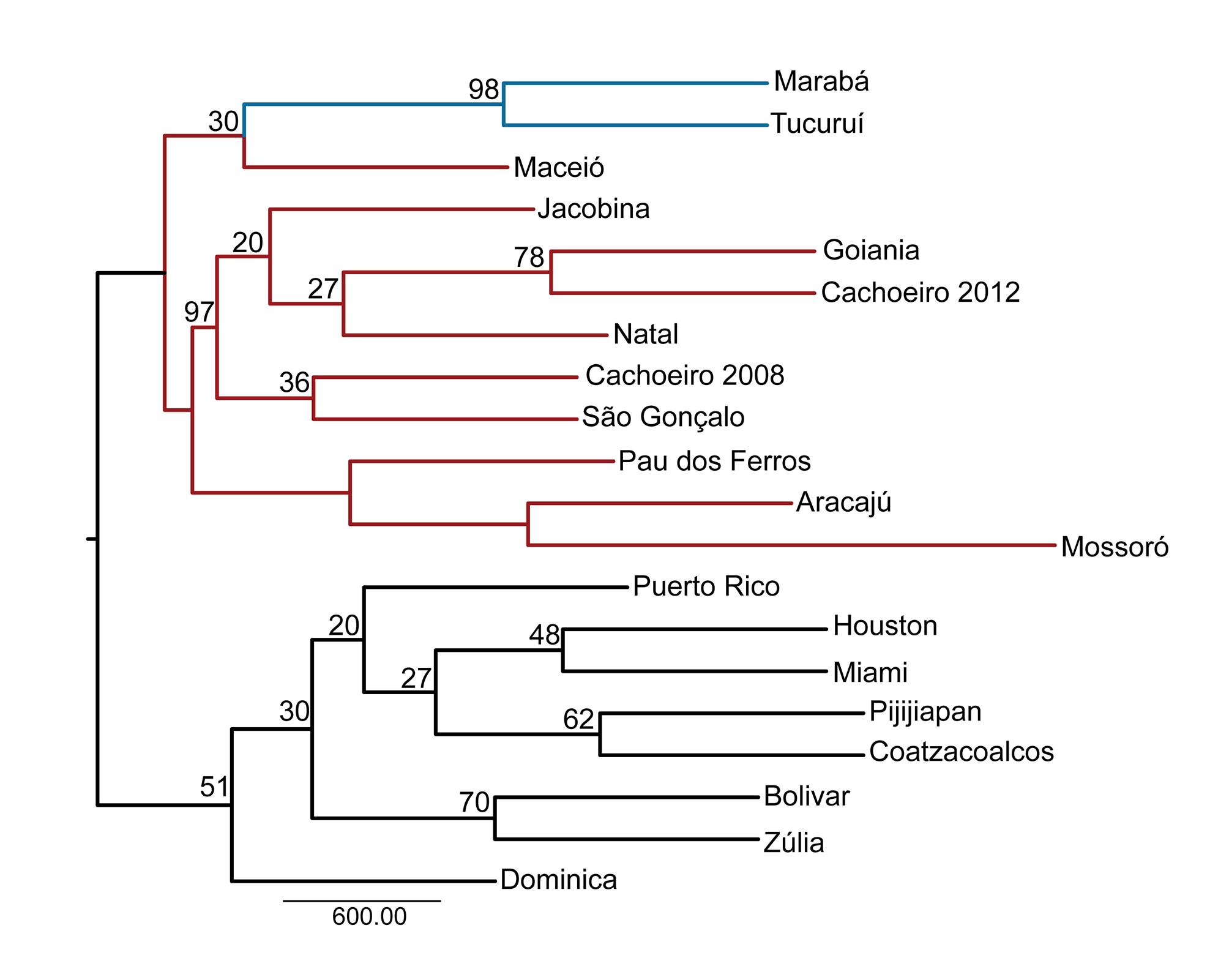

Supplement: Figure S1 — Neighbor-joining tree based on Cavalli-Sforza and Edward's chord distance computed from the allele frequencies of the 12 microsatellite loci analyzed. Unrooted tree. Numbers on branches indicate bootstrap values from 1000 replicates. Colors correspond to K = 2 cluster analysis displayed on figure 2. (TIF) [file pntd.0003167.s001.tif]

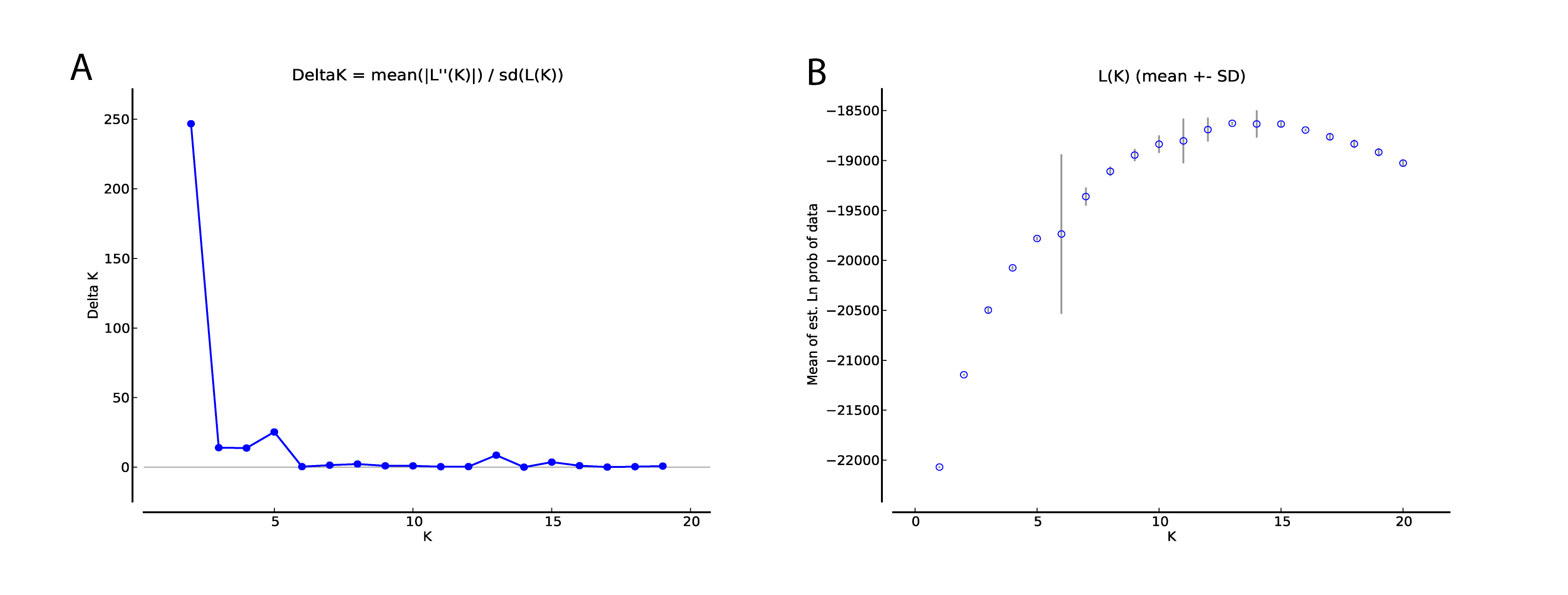

Supplement: Figure S2 — Scatter plots of ΔK (A) and Log probability of the data (B) for all Ae. aegypti populations analyzed. ΔK plots are based on the rate of change in the log probability of the data between successive K values. (TIF) [file pntd.0003167.s002.tif]

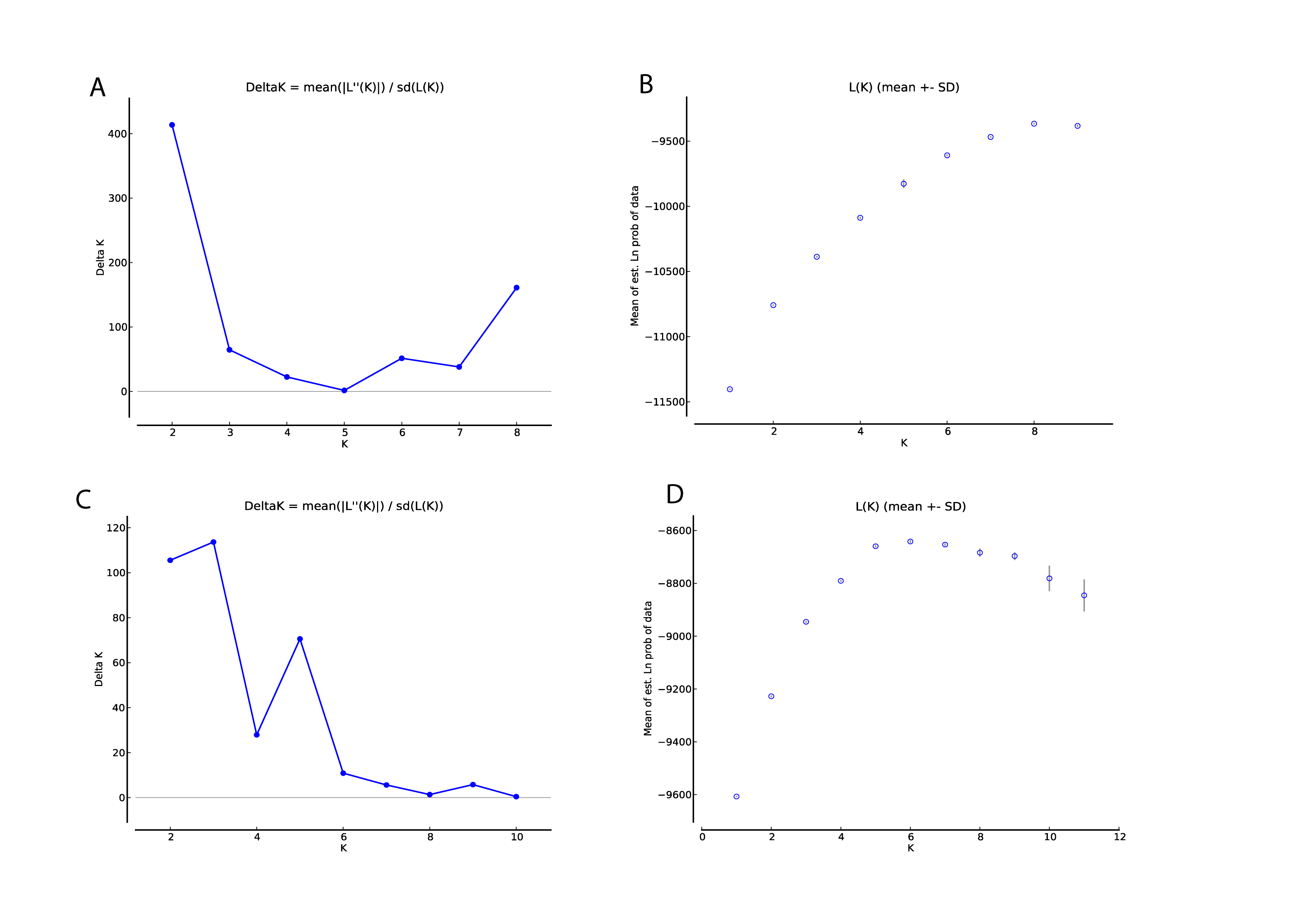

Supplement: Figure S3 — Scatter plots of ΔK (A and C) and Log probability of the data (B and D) for the two groups of Ae. aegypti populations. ΔK plots are based on the rate of change in the log probability of the data between successive K values. A and B – blue group from STRUCTURE analysis on figure 2 (Tucuruí, Marabá, USA, Mexico, Puerto Rico and Venezuela), C and D – red group from STRUCTURE analysis on figure 2 (Dominica and all other Brazilian populations except Tucuruí and Marabá). (TIF) [file pntd.0003167.s003.tif]
