# Supplementary material for: Genetic Diversity of Brazilian Aedes aegypti: Patterns following an Eradication Program
Source: PLoS Negl Trop Dis. 2014 Sep 18;8(9):e3167. doi: 10.1371/journal.pntd.0003167 (PMC4169244; doi:10.1371/journal.pntd.0003167)
Supplement: Table S1 — Aedes aegypti FIS values by locus. (DOC) [file pntd.0003167.s006.doc]

Table S1. *Aedes aegypti* F_IS_ values by Locus. Values significant after sequential Bonferroni correction are marked in bold font.

|  | **Loci Names** | | | | | | | | | | | |
| --- | --- | --- | --- | --- | --- | --- | --- | --- | --- | --- | --- | --- |
| **Population** | **AC1** | **AC2** | **AC4** | **AC5** | **CT2** | **AG1** | **AG2** | **AG5** | **A1** | **A9** | **B2** | **B3** |
| Aracaju | -0.1702 | 0.0303 | -0.1396 | 0.3716 | -0.0222 | -0.0534 | 0.0987 | -0.0455 | -0.1237 | 0.2879 | -0.0222 | -0.0952 |
| Goiania | 0.0022 | 0.1218 | -0.1795 | **0.444** | 0.4027 | -0.1157 | 0.0707 | -0.0474 | -0.3604 | 0.4483 | -0.15 | 0.428 |
| Maceio | -0.3248 | -0.2655 | -0.0952 | 0.4524 | -0.0222 | 0.0174 | -0.1247 | 0.1831 | -0.0845 | **0.8544** | -0.122 | 0.3224 |
| Mossoro | -0.0278 | 0.3347 | -0.2618 | 0.0803 | -0.0769 | 0.0222 | 0.0597 | -0.2353 | -0.2264 | 0.4366 | NA | -0.0338 |
| Pau Ferros | 0.0308 | -0.0687 | -0.1887 | 0.5536 | -0.0056 | -0.0496 | 0.3077 | -0.0526 | 0.2708 | 0.6757 | NA | 0.6104 |
| Tucuruí | -0.2923 | -0.1915 | -0.8 | **0.3361** | -0.0136 | -0.3678 | 0.0867 | 0.0024 | -0.1271 | -0.2367 | NA | 0.069 |
| Marabá | 0.0928 | 0.1345 | 0.2385 | -0.2078 | 0.0877 | 0.049 | 0.1877 | 0.0807 | 0.0827 | 0.2295 | 0.0864 | -0.2447 |
| Cachoeiro 2008 | -0.0584 | -0.2044 | -0.1282 | 0.0833 | 0.0571 | -0.1231 | 0.2831 | -0.2571 | -0.068 | **0.6765** | -0.0476 | 0.033 |
| Cachoeiro 2012 | -0.0208 | 0.2436 | -0.12 | 0.1269 | 0.3062 | -0.0375 | -0.1313 | 0.1683 | -0.1421 | 0.313 | -0.0633 | 0.0776 |
| Jacobina | 0.1711 | -0.1131 | -0.0101 | -0.0564 | 0.1628 | -0.1247 | 0.0475 | -0.1059 | 0.08 | **0.4285** | 0.0698 | 0.2664 |
| Natal | -0.0208 | -0.0725 | -0.1084 | 0.0297 | -0.0455 | 0.08 | -0.0375 | -0.0521 | -0.1543 | 0.2048 | 0.0841 | 0.0582 |
| São Gonçalo | -0.0385 | 0.1076 | -0.3103 | -0.3725 | 0.0221 | 0.2197 | 0.1633 | -0.0253 | 0.0144 | **0.9086** | -0.01 | -0.1343 |
| Bolivar, Venezuela | 0.3136 | 0.0385 | -0.1059 | 0.2231 | -0.089 | -0.087 | 0.0188 | 0.0868 | 0.234 | 0.0996 | -0.2963 | -0.0281 |
| Zulia, Venezuela | -0.1025 | -0.1804 | 0.0036 | 0.006 | -0.1327 | -0.1341 | 0.0953 | 0.0351 | 0.0054 | -0.0891 | -0.1358 | 0.102 |
| Houston, TX, USA | -0.2027 | 0.7431 | -0.1216 | **-0.4308** | -0.4 | 0.0903 | -0.1933 | -0.2905 | -0.28 | -0.1064 | NA | -0.0629 |
| Coatzacoalcos, Mexico | -0.3606 | 0.0889 | -0.0889 | -0.1867 | NA | -0.2575 | -0.2899 | -0.0769 | **-0.3236** | -0.0652 | NA | -0.1109 |
| Pijijiapan, Mexico | 0.2957 | 0.1431 | 0.2384 | 0.0439 | NA | **0.5007** | 0.1298 | -0.0305 | -0.074 | 0.3192 | -0.011 | 0.0438 |
| Dominica | -0.0261 | -0.0836 | 0.6506 | 0.0258 | NA | -0.0749 | **0.251** | -0.1542 | -0.133 | **0.621** | NA | 0.1056 |
| Puerto Rico | 0.0356 | -0.12 | -0.15 | 0.0173 | -0.1084 | 0.0733 | 0.1211 | 0.0079 | -0.0153 | 0.2471 | -0.0455 | 0.0057 |
| Miami, Florida, USA | -0.0568 | -0.1717 | -0.3978 | -0.1135 | -0.0583 | 0.0775 | -0.1663 | 0.0543 | -0.1368 | 0.2637 | -0.122 | -0.0035 |
