# Supplementary material for: Genetic Diversity of Brazilian Aedes aegypti: Patterns following an Eradication Program
Source: PLoS Negl Trop Dis. 2014 Sep 18;8(9):e3167. doi: 10.1371/journal.pntd.0003167 (PMC4169244; doi:10.1371/journal.pntd.0003167)
Supplement: Table S2 — MicroChecker v2.2.3 null allele frequency. (DOC) [file pntd.0003167.s007.doc]

Table S2. MicroChecker v2.2.3 null allele frequency.

| **Population/Loci** | **A9** | **AG2** | **AC4** | **AG1** | **AC1** | **AC2** | **AC5** | **B3** |
| --- | --- | --- | --- | --- | --- | --- | --- | --- |
| **Miami** | 0.1005 | - | - | - | - | - | - | - |
| **Puerto Rico** | 0.0995 | - | - | - | - | - | - | - |
| **Dominica** | 0.2291 | 0.0907 | 0.0676 | - | - | - | - | - |
| **Pijijiapan** | 0.1217 | - | - | 0.2003 | 0.1034 | - | - | - |
| **Houston** | - | - | - | - | - | 0.0847 | - | - |
| **Bolivar** | - | - | - | - | 0.1208 | - | 0.0907 | - |
| **São Gonçalo** | 0.3196 | - | - | - | - | - | - | - |
| **Marabá** | 0.0835 | 0.0738 | - | - | - | - | - | - |
| **Tucuruí** | - | - | - | - | - | - | 0.1271 | - |
| **Paus dos Ferros** | 0.2610 | 0.1251 | - | - | - | - | 0.2123 | - |
| **Maceió** | 0.3174 | - | - | - | - | - | 0.0992 | - |
| **Cachoeiro 2008** | 0.2267 | 0.0843 | - | - | - | - | - | - |
| **Jacobina** | 0.1687 | - | - | - | 0.0567 | - | - | 0.0660 |
| **Goiania** | 0.1429 | - | - | - | - | - | 0.1896 | 0.1430 |
