# Supplementary material for: Genetic Diversity of Brazilian Aedes aegypti: Patterns following an Eradication Program
Source: PLoS Negl Trop Dis. 2014 Sep 18;8(9):e3167. doi: 10.1371/journal.pntd.0003167 (PMC4169244; doi:10.1371/journal.pntd.0003167)
Supplement: Table S3 — Allele frequencies for the 12 microsatellite loci analyzed. (DOC) [file pntd.0003167.s008.doc]

Table S3. Allele Frequencies for the 12 microsatellite loci analyzed. Locus name, allele name, number of individuals genotyped for each loci (N), HP-rare allelic richness (Na), HP-rare private allelic richness (Np), Observed heterozigosity (Ho), Expected unbiased heterozigosity (He) for each population.

| **Locus** | **Allele** | **Aracajú** | **Goiania** | **Maceió** | **Mossoró** | **Pau Ferros** | **Tucuruí** | **Marabá** | **Cachoeiro 2008** | **Cachoeiro 2012** | **Jacobina** | **Natal** | **São Gonçalo** | **Bolivar** | **Zulia** | **Huston** | **Coatzacoalcos** | **Pijijiapan** | **Dominica** | **Puerto_Rico** | **Miami** |
| --- | --- | --- | --- | --- | --- | --- | --- | --- | --- | --- | --- | --- | --- | --- | --- | --- | --- | --- | --- | --- | --- |
| **AC1** | **N** | 23 | 24 | 24 | 21 | 15 | 15 | 48 | 23 | 43 | 92 | 47 | 19 | 48 | 47 | 29 | 50 | 47 | 48 | 47 | 47 |
|  | **195** | 0.000 | 0.021 | 0.000 | 0.024 | 0.067 | 0.000 | 0.010 | 0.065 | 0.000 | 0.011 | 0.011 | 0.132 | 0.021 | 0.064 | 0.362 | 0.320 | 0.085 | 0.000 | 0.191 | 0.245 |
|  | **197** | 0.543 | 0.083 | 0.479 | 0.357 | 0.533 | 0.400 | 0.281 | 0.370 | 0.186 | 0.201 | 0.330 | 0.289 | 0.292 | 0.372 | 0.086 | 0.000 | 0.394 | 0.219 | 0.170 | 0.351 |
|  | **199** | 0.000 | 0.000 | 0.000 | 0.000 | 0.033 | 0.133 | 0.115 | 0.000 | 0.000 | 0.000 | 0.000 | 0.000 | 0.000 | 0.000 | 0.000 | 0.000 | 0.000 | 0.000 | 0.000 | 0.000 |
|  | **201** | 0.065 | 0.146 | 0.021 | 0.048 | 0.033 | 0.000 | 0.031 | 0.000 | 0.174 | 0.141 | 0.223 | 0.000 | 0.188 | 0.106 | 0.241 | 0.120 | 0.000 | 0.281 | 0.074 | 0.021 |
|  | **207** | 0.000 | 0.000 | 0.000 | 0.000 | 0.000 | 0.000 | 0.000 | 0.022 | 0.012 | 0.000 | 0.000 | 0.026 | 0.031 | 0.011 | 0.000 | 0.000 | 0.000 | 0.000 | 0.000 | 0.000 |
|  | **209** | 0.391 | 0.750 | 0.458 | 0.571 | 0.333 | 0.467 | 0.563 | 0.543 | 0.628 | 0.647 | 0.426 | 0.553 | 0.469 | 0.394 | 0.310 | 0.560 | 0.521 | 0.500 | 0.564 | 0.372 |
|  | **211** | 0.000 | 0.000 | 0.000 | 0.000 | 0.000 | 0.000 | 0.000 | 0.000 | 0.000 | 0.000 | 0.011 | 0.000 | 0.000 | 0.053 | 0.000 | 0.000 | 0.000 | 0.000 | 0.000 | 0.011 |
|  | **215** | 0.000 | 0.000 | 0.042 | 0.000 | 0.000 | 0.000 | 0.000 | 0.000 | 0.000 | 0.000 | 0.000 | 0.000 | 0.000 | 0.000 | 0.000 | 0.000 | 0.000 | 0.000 | 0.000 | 0.000 |
| **AC2** | **N** | 21 | 21 | 24 | 22 | 15 | 17 | 48 | 23 | 43 | 92 | 47 | 20 | 48 | 47 | 29 | 50 | 47 | 48 | 47 | 47 |
|  | **180** | 0.000 | 0.000 | 0.000 | 0.000 | 0.000 | 0.000 | 0.000 | 0.000 | 0.000 | 0.000 | 0.000 | 0.050 | 0.000 | 0.000 | 0.000 | 0.000 | 0.000 | 0.000 | 0.000 | 0.000 |
|  | **182** | 0.000 | 0.000 | 0.000 | 0.000 | 0.000 | 0.000 | 0.000 | 0.000 | 0.000 | 0.000 | 0.000 | 0.000 | 0.000 | 0.000 | 0.000 | 0.000 | 0.000 | 0.000 | 0.000 | 0.021 |
|  | **184** | 0.119 | 0.238 | 0.250 | 0.341 | 0.267 | 0.176 | 0.229 | 0.217 | 0.174 | 0.125 | 0.489 | 0.425 | 0.135 | 0.160 | 0.017 | 0.010 | 0.043 | 0.000 | 0.638 | 0.202 |
|  | **186** | 0.333 | 0.571 | 0.313 | 0.068 | 0.200 | 0.029 | 0.052 | 0.152 | 0.174 | 0.136 | 0.032 | 0.150 | 0.083 | 0.011 | 0.052 | 0.210 | 0.489 | 0.000 | 0.170 | 0.096 |
|  | **188** | 0.548 | 0.190 | 0.438 | 0.591 | 0.533 | 0.794 | 0.719 | 0.630 | 0.651 | 0.734 | 0.479 | 0.375 | 0.781 | 0.830 | 0.931 | 0.780 | 0.468 | 0.813 | 0.191 | 0.681 |
|  | **190** | 0.000 | 0.000 | 0.000 | 0.000 | 0.000 | 0.000 | 0.000 | 0.000 | 0.000 | 0.005 | 0.000 | 0.000 | 0.000 | 0.000 | 0.000 | 0.000 | 0.000 | 0.188 | 0.000 | 0.000 |
| **AC4** | **N** | 24 | 24 | 24 | 22 | 15 | 19 | 48 | 23 | 43 | 92 | 47 | 20 | 48 | 47 | 29 | 50 | 47 | 48 | 47 | 47 |
|  | **128** | 0.729 | 0.583 | 0.896 | 0.545 | 0.567 | 0.447 | 0.583 | 0.870 | 0.884 | 0.761 | 0.553 | 0.750 | 0.896 | 0.255 | 0.707 | 0.910 | 0.926 | 0.938 | 0.862 | 0.479 |
|  | **130** | 0.271 | 0.417 | 0.104 | 0.455 | 0.433 | 0.553 | 0.417 | 0.130 | 0.116 | 0.239 | 0.447 | 0.250 | 0.104 | 0.745 | 0.276 | 0.090 | 0.074 | 0.063 | 0.138 | 0.521 |
|  | **134** | 0.000 | 0.000 | 0.000 | 0.000 | 0.000 | 0.000 | 0.000 | 0.000 | 0.000 | 0.000 | 0.000 | 0.000 | 0.000 | 0.000 | 0.017 | 0.000 | 0.000 | 0.000 | 0.000 | 0.000 |
| **CT2** | **N** | 24 | 23 | 24 | 22 | 16 | 18 | 48 | 23 | 43 | 92 | 47 | 20 | 48 | 47 | 29 | 50 | 47 | 48 | 47 | 46 |
|  | **184** | 0.958 | 0.696 | 0.958 | 0.909 | 0.781 | 0.500 | 0.427 | 0.717 | 0.651 | 0.908 | 0.947 | 0.775 | 0.385 | 0.213 | 0.707 | 1.000 | 1.000 | 0.990 | 0.702 | 0.641 |
|  | **188** | 0.042 | 0.304 | 0.042 | 0.091 | 0.156 | 0.389 | 0.469 | 0.283 | 0.337 | 0.092 | 0.053 | 0.225 | 0.615 | 0.787 | 0.293 | 0.000 | 0.000 | 0.010 | 0.298 | 0.348 |
|  | **190** | 0.000 | 0.000 | 0.000 | 0.000 | 0.063 | 0.000 | 0.000 | 0.000 | 0.000 | 0.000 | 0.000 | 0.000 | 0.000 | 0.000 | 0.000 | 0.000 | 0.000 | 0.000 | 0.000 | 0.000 |
|  | **194** | 0.000 | 0.000 | 0.000 | 0.000 | 0.000 | 0.111 | 0.104 | 0.000 | 0.012 | 0.000 | 0.000 | 0.000 | 0.000 | 0.000 | 0.000 | 0.000 | 0.000 | 0.000 | 0.000 | 0.011 |
| **AG1** | **N** | 24 | 22 | 24 | 21 | 16 | 18 | 48 | 23 | 43 | 92 | 47 | 20 | 48 | 47 | 29 | 50 | 46 | 48 | 47 | 47 |
|  | **113** | 0.000 | 0.000 | 0.000 | 0.000 | 0.000 | 0.000 | 0.000 | 0.000 | 0.000 | 0.027 | 0.000 | 0.000 | 0.000 | 0.000 | 0.000 | 0.000 | 0.000 | 0.000 | 0.000 | 0.000 |
|  | **115** | 0.146 | 0.000 | 0.146 | 0.071 | 0.219 | 0.083 | 0.188 | 0.000 | 0.012 | 0.000 | 0.160 | 0.000 | 0.208 | 0.117 | 0.017 | 0.250 | 0.326 | 0.031 | 0.053 | 0.223 |
|  | **117** | 0.104 | 0.068 | 0.313 | 0.238 | 0.250 | 0.028 | 0.052 | 0.413 | 0.186 | 0.625 | 0.362 | 0.350 | 0.375 | 0.564 | 0.000 | 0.060 | 0.174 | 0.615 | 0.479 | 0.287 |
|  | **119** | 0.146 | 0.500 | 0.083 | 0.048 | 0.063 | 0.306 | 0.354 | 0.391 | 0.419 | 0.076 | 0.138 | 0.175 | 0.146 | 0.053 | 0.190 | 0.690 | 0.087 | 0.146 | 0.245 | 0.223 |
|  | **121** | 0.604 | 0.432 | 0.458 | 0.643 | 0.438 | 0.583 | 0.406 | 0.174 | 0.384 | 0.272 | 0.340 | 0.475 | 0.271 | 0.266 | 0.793 | 0.000 | 0.413 | 0.208 | 0.223 | 0.255 |
|  | **123** | 0.000 | 0.000 | 0.000 | 0.000 | 0.031 | 0.000 | 0.000 | 0.022 | 0.000 | 0.000 | 0.000 | 0.000 | 0.000 | 0.000 | 0.000 | 0.000 | 0.000 | 0.000 | 0.000 | 0.011 |
| **AG2** | **N** | 22 | 24 | 24 | 20 | 15 | 19 | 48 | 23 | 43 | 92 | 47 | 20 | 48 | 47 | 29 | 50 | 47 | 48 | 47 | 43 |
|  | **114** | 0.000 | 0.000 | 0.000 | 0.000 | 0.000 | 0.000 | 0.000 | 0.000 | 0.000 | 0.000 | 0.000 | 0.000 | 0.052 | 0.000 | 0.000 | 0.000 | 0.000 | 0.000 | 0.000 | 0.023 |
|  | **115** | 0.091 | 0.021 | 0.063 | 0.075 | 0.067 | 0.158 | 0.229 | 0.065 | 0.058 | 0.326 | 0.000 | 0.425 | 0.458 | 0.245 | 0.741 | 0.560 | 0.585 | 0.573 | 0.340 | 0.372 |
|  | **117** | 0.045 | 0.313 | 0.000 | 0.025 | 0.100 | 0.105 | 0.021 | 0.043 | 0.151 | 0.152 | 0.223 | 0.000 | 0.177 | 0.245 | 0.224 | 0.120 | 0.000 | 0.000 | 0.138 | 0.186 |
|  | **119** | 0.477 | 0.438 | 0.792 | 0.250 | 0.267 | 0.526 | 0.469 | 0.717 | 0.326 | 0.239 | 0.415 | 0.325 | 0.083 | 0.351 | 0.000 | 0.000 | 0.000 | 0.229 | 0.160 | 0.128 |
|  | **129** | 0.000 | 0.000 | 0.000 | 0.000 | 0.000 | 0.000 | 0.010 | 0.000 | 0.000 | 0.000 | 0.000 | 0.000 | 0.000 | 0.000 | 0.034 | 0.000 | 0.000 | 0.000 | 0.000 | 0.058 |
|  | **131** | 0.000 | 0.000 | 0.000 | 0.025 | 0.000 | 0.105 | 0.042 | 0.000 | 0.000 | 0.016 | 0.000 | 0.000 | 0.000 | 0.043 | 0.000 | 0.000 | 0.351 | 0.000 | 0.032 | 0.058 |
|  | **133** | 0.000 | 0.000 | 0.000 | 0.025 | 0.000 | 0.000 | 0.000 | 0.000 | 0.000 | 0.000 | 0.000 | 0.000 | 0.000 | 0.000 | 0.000 | 0.000 | 0.011 | 0.000 | 0.064 | 0.000 |
|  | **135** | 0.000 | 0.000 | 0.000 | 0.000 | 0.000 | 0.053 | 0.115 | 0.000 | 0.000 | 0.000 | 0.021 | 0.000 | 0.000 | 0.000 | 0.000 | 0.320 | 0.053 | 0.000 | 0.117 | 0.000 |
|  | **137** | 0.000 | 0.000 | 0.000 | 0.000 | 0.000 | 0.000 | 0.042 | 0.000 | 0.000 | 0.000 | 0.000 | 0.000 | 0.010 | 0.000 | 0.000 | 0.000 | 0.000 | 0.000 | 0.064 | 0.000 |
|  | **139** | 0.000 | 0.042 | 0.042 | 0.000 | 0.100 | 0.000 | 0.000 | 0.000 | 0.000 | 0.000 | 0.000 | 0.000 | 0.000 | 0.000 | 0.000 | 0.000 | 0.000 | 0.104 | 0.000 | 0.012 |
|  | **141** | 0.000 | 0.000 | 0.000 | 0.000 | 0.000 | 0.000 | 0.000 | 0.000 | 0.000 | 0.000 | 0.000 | 0.000 | 0.000 | 0.000 | 0.000 | 0.000 | 0.000 | 0.042 | 0.000 | 0.000 |
|  | **143** | 0.068 | 0.021 | 0.000 | 0.000 | 0.000 | 0.000 | 0.021 | 0.000 | 0.035 | 0.011 | 0.011 | 0.000 | 0.010 | 0.000 | 0.000 | 0.000 | 0.000 | 0.052 | 0.021 | 0.047 |
|  | **145** | 0.023 | 0.000 | 0.000 | 0.000 | 0.000 | 0.000 | 0.021 | 0.000 | 0.012 | 0.022 | 0.000 | 0.000 | 0.010 | 0.106 | 0.000 | 0.000 | 0.000 | 0.000 | 0.053 | 0.012 |
|  | **149** | 0.000 | 0.021 | 0.000 | 0.000 | 0.000 | 0.000 | 0.000 | 0.000 | 0.000 | 0.000 | 0.000 | 0.000 | 0.000 | 0.011 | 0.000 | 0.000 | 0.000 | 0.000 | 0.011 | 0.000 |
|  | **151** | 0.000 | 0.000 | 0.000 | 0.000 | 0.000 | 0.000 | 0.000 | 0.000 | 0.000 | 0.000 | 0.000 | 0.000 | 0.073 | 0.000 | 0.000 | 0.000 | 0.000 | 0.000 | 0.000 | 0.105 |
|  | **153** | 0.000 | 0.000 | 0.000 | 0.000 | 0.100 | 0.000 | 0.010 | 0.000 | 0.000 | 0.000 | 0.021 | 0.000 | 0.000 | 0.000 | 0.000 | 0.000 | 0.000 | 0.000 | 0.000 | 0.000 |
|  | **155** | 0.000 | 0.042 | 0.021 | 0.075 | 0.000 | 0.026 | 0.021 | 0.000 | 0.012 | 0.005 | 0.106 | 0.000 | 0.052 | 0.000 | 0.000 | 0.000 | 0.000 | 0.000 | 0.000 | 0.000 |
|  | **157** | 0.023 | 0.063 | 0.000 | 0.050 | 0.100 | 0.026 | 0.000 | 0.065 | 0.174 | 0.027 | 0.032 | 0.050 | 0.073 | 0.000 | 0.000 | 0.000 | 0.000 | 0.000 | 0.000 | 0.000 |
|  | **159** | 0.068 | 0.021 | 0.000 | 0.075 | 0.000 | 0.000 | 0.000 | 0.022 | 0.128 | 0.033 | 0.000 | 0.100 | 0.000 | 0.000 | 0.000 | 0.000 | 0.000 | 0.000 | 0.000 | 0.000 |
|  | **161** | 0.068 | 0.021 | 0.000 | 0.000 | 0.033 | 0.000 | 0.000 | 0.000 | 0.000 | 0.016 | 0.000 | 0.000 | 0.000 | 0.000 | 0.000 | 0.000 | 0.000 | 0.000 | 0.000 | 0.000 |
|  | **163** | 0.023 | 0.000 | 0.042 | 0.050 | 0.000 | 0.000 | 0.000 | 0.000 | 0.000 | 0.000 | 0.000 | 0.050 | 0.000 | 0.000 | 0.000 | 0.000 | 0.000 | 0.000 | 0.000 | 0.000 |
|  | **165** | 0.000 | 0.000 | 0.000 | 0.000 | 0.000 | 0.000 | 0.000 | 0.000 | 0.000 | 0.005 | 0.000 | 0.000 | 0.000 | 0.000 | 0.000 | 0.000 | 0.000 | 0.000 | 0.000 | 0.000 |
|  | **167** | 0.091 | 0.000 | 0.000 | 0.125 | 0.233 | 0.000 | 0.000 | 0.043 | 0.047 | 0.125 | 0.043 | 0.050 | 0.000 | 0.000 | 0.000 | 0.000 | 0.000 | 0.000 | 0.000 | 0.000 |
|  | **169** | 0.023 | 0.000 | 0.000 | 0.075 | 0.000 | 0.000 | 0.000 | 0.022 | 0.058 | 0.022 | 0.106 | 0.000 | 0.000 | 0.000 | 0.000 | 0.000 | 0.000 | 0.000 | 0.000 | 0.000 |
|  | **171** | 0.000 | 0.000 | 0.042 | 0.100 | 0.000 | 0.000 | 0.000 | 0.000 | 0.000 | 0.000 | 0.021 | 0.000 | 0.000 | 0.000 | 0.000 | 0.000 | 0.000 | 0.000 | 0.000 | 0.000 |
|  | **173** | 0.000 | 0.000 | 0.000 | 0.050 | 0.000 | 0.000 | 0.000 | 0.022 | 0.000 | 0.000 | 0.000 | 0.000 | 0.000 | 0.000 | 0.000 | 0.000 | 0.000 | 0.000 | 0.000 | 0.000 |
| **AG5** | **N** | 24 | 24 | 24 | 22 | 16 | 18 | 48 | 23 | 43 | 92 | 47 | 20 | 48 | 47 | 29 | 50 | 47 | 48 | 47 | 47 |
|  | **166** | 0.146 | 0.000 | 0.125 | 0.000 | 0.000 | 0.056 | 0.073 | 0.000 | 0.035 | 0.005 | 0.011 | 0.050 | 0.385 | 0.117 | 0.000 | 0.000 | 0.000 | 0.073 | 0.223 | 0.053 |
|  | **168** | 0.146 | 0.708 | 0.042 | 0.205 | 0.188 | 0.139 | 0.167 | 0.196 | 0.465 | 0.413 | 0.511 | 0.250 | 0.219 | 0.096 | 0.310 | 0.000 | 0.479 | 0.156 | 0.181 | 0.149 |
|  | **170** | 0.000 | 0.000 | 0.000 | 0.000 | 0.000 | 0.000 | 0.031 | 0.000 | 0.012 | 0.000 | 0.000 | 0.125 | 0.000 | 0.000 | 0.293 | 0.000 | 0.000 | 0.000 | 0.011 | 0.085 |
|  | **172** | 0.000 | 0.063 | 0.021 | 0.000 | 0.031 | 0.000 | 0.031 | 0.022 | 0.058 | 0.005 | 0.043 | 0.025 | 0.083 | 0.245 | 0.190 | 0.080 | 0.043 | 0.042 | 0.032 | 0.213 |
|  | **174** | 0.021 | 0.000 | 0.000 | 0.000 | 0.000 | 0.000 | 0.000 | 0.000 | 0.000 | 0.000 | 0.000 | 0.025 | 0.000 | 0.000 | 0.000 | 0.000 | 0.000 | 0.229 | 0.149 | 0.032 |
|  | **176** | 0.563 | 0.125 | 0.458 | 0.795 | 0.594 | 0.444 | 0.302 | 0.717 | 0.326 | 0.484 | 0.394 | 0.375 | 0.198 | 0.213 | 0.000 | 0.920 | 0.255 | 0.354 | 0.085 | 0.160 |
|  | **178** | 0.125 | 0.104 | 0.354 | 0.000 | 0.188 | 0.361 | 0.396 | 0.065 | 0.105 | 0.092 | 0.043 | 0.125 | 0.115 | 0.330 | 0.207 | 0.000 | 0.223 | 0.146 | 0.319 | 0.309 |
|  | **180** | 0.000 | 0.000 | 0.000 | 0.000 | 0.000 | 0.000 | 0.000 | 0.000 | 0.000 | 0.000 | 0.000 | 0.025 | 0.000 | 0.000 | 0.000 | 0.000 | 0.000 | 0.000 | 0.000 | 0.000 |
| **A1** | **N** | 24 | 24 | 24 | 21 | 15 | 18 | 48 | 23 | 43 | 92 | 47 | 20 | 48 | 47 | 29 | 50 | 47 | 48 | 47 | 47 |
|  | **156** | 0.000 | 0.354 | 0.042 | 0.000 | 0.000 | 0.139 | 0.115 | 0.000 | 0.093 | 0.098 | 0.085 | 0.000 | 0.000 | 0.000 | 0.000 | 0.310 | 0.000 | 0.000 | 0.000 | 0.160 |
|  | **158** | 0.104 | 0.021 | 0.000 | 0.000 | 0.000 | 0.028 | 0.000 | 0.065 | 0.023 | 0.038 | 0.000 | 0.050 | 0.385 | 0.191 | 0.603 | 0.180 | 0.479 | 0.115 | 0.245 | 0.234 |
|  | **159** | 0.000 | 0.000 | 0.000 | 0.000 | 0.000 | 0.000 | 0.000 | 0.000 | 0.000 | 0.000 | 0.000 | 0.000 | 0.000 | 0.000 | 0.000 | 0.000 | 0.213 | 0.000 | 0.011 | 0.149 |
|  | **162** | 0.396 | 0.000 | 0.271 | 0.452 | 0.033 | 0.250 | 0.292 | 0.043 | 0.128 | 0.185 | 0.223 | 0.250 | 0.531 | 0.245 | 0.310 | 0.000 | 0.053 | 0.260 | 0.128 | 0.021 |
|  | **168** | 0.500 | 0.625 | 0.688 | 0.548 | 0.700 | 0.583 | 0.583 | 0.891 | 0.756 | 0.679 | 0.670 | 0.700 | 0.073 | 0.394 | 0.000 | 0.200 | 0.085 | 0.625 | 0.521 | 0.170 |
|  | **171** | 0.000 | 0.000 | 0.000 | 0.000 | 0.267 | 0.000 | 0.010 | 0.000 | 0.000 | 0.000 | 0.021 | 0.000 | 0.010 | 0.170 | 0.086 | 0.310 | 0.170 | 0.000 | 0.096 | 0.266 |
| **B2** | **N** | 24 | 24 | 24 | 22 | 16 | 19 | 48 | 23 | 43 | 92 | 47 | 20 | 48 | 47 | 29 | 50 | 47 | 48 | 47 | 47 |
|  | **95** | 0.042 | 0.146 | 0.125 | 0.000 | 0.000 | 0.000 | 0.115 | 0.065 | 0.186 | 0.152 | 0.117 | 0.225 | 0.000 | 0.000 | 0.000 | 0.000 | 0.000 | 0.000 | 0.000 | 0.117 |
|  | **107** | 0.000 | 0.000 | 0.000 | 0.000 | 0.000 | 0.000 | 0.000 | 0.000 | 0.000 | 0.005 | 0.000 | 0.025 | 0.448 | 0.128 | 0.017 | 0.000 | 0.021 | 0.000 | 0.053 | 0.000 |
|  | **113** | 0.958 | 0.854 | 0.875 | 1.000 | 1.000 | 1.000 | 0.885 | 0.935 | 0.814 | 0.842 | 0.883 | 0.750 | 0.552 | 0.872 | 0.983 | 1.000 | 0.979 | 1.000 | 0.947 | 0.883 |
| **B3** | **N** | 24 | 22 | 24 | 22 | 16 | 19 | 48 | 23 | 43 | 92 | 47 | 20 | 48 | 47 | 29 | 50 | 47 | 48 | 47 | 47 |
|  | **148** | 0.000 | 0.000 | 0.000 | 0.000 | 0.000 | 0.000 | 0.000 | 0.000 | 0.000 | 0.033 | 0.000 | 0.000 | 0.021 | 0.011 | 0.000 | 0.000 | 0.138 | 0.250 | 0.053 | 0.011 |
|  | **160** | 0.000 | 0.000 | 0.000 | 0.000 | 0.000 | 0.000 | 0.000 | 0.000 | 0.000 | 0.000 | 0.000 | 0.050 | 0.000 | 0.000 | 0.000 | 0.000 | 0.000 | 0.000 | 0.000 | 0.000 |
|  | **166** | 0.896 | 0.523 | 0.500 | 0.795 | 0.813 | 0.553 | 0.615 | 0.848 | 0.791 | 0.804 | 0.787 | 0.800 | 0.740 | 0.649 | 0.517 | 0.090 | 0.745 | 0.167 | 0.426 | 0.532 |
|  | **172** | 0.104 | 0.045 | 0.458 | 0.068 | 0.000 | 0.447 | 0.375 | 0.130 | 0.058 | 0.092 | 0.096 | 0.100 | 0.083 | 0.117 | 0.172 | 0.860 | 0.085 | 0.135 | 0.298 | 0.340 |
|  | **175** | 0.000 | 0.432 | 0.042 | 0.136 | 0.188 | 0.000 | 0.010 | 0.022 | 0.151 | 0.071 | 0.117 | 0.050 | 0.156 | 0.223 | 0.310 | 0.050 | 0.032 | 0.448 | 0.223 | 0.117 |
| **AC5** | **N** | 24 | 24 | 24 | 22 | 16 | 19 | 48 | 23 | 43 | 92 | 47 | 20 | 48 | 47 | 29 | 50 | 47 | 48 | 46 | 47 |
|  | **147** | 0.000 | 0.104 | 0.042 | 0.000 | 0.000 | 0.000 | 0.000 | 0.000 | 0.023 | 0.000 | 0.064 | 0.000 | 0.063 | 0.085 | 0.121 | 0.000 | 0.000 | 0.000 | 0.033 | 0.191 |
|  | **148** | 0.000 | 0.083 | 0.000 | 0.000 | 0.000 | 0.000 | 0.000 | 0.000 | 0.000 | 0.000 | 0.000 | 0.000 | 0.000 | 0.000 | 0.000 | 0.000 | 0.000 | 0.000 | 0.000 | 0.011 |
|  | **151** | 0.000 | 0.000 | 0.000 | 0.000 | 0.000 | 0.000 | 0.000 | 0.022 | 0.000 | 0.000 | 0.000 | 0.000 | 0.000 | 0.000 | 0.000 | 0.000 | 0.000 | 0.000 | 0.000 | 0.000 |
|  | **152** | 0.000 | 0.000 | 0.000 | 0.000 | 0.063 | 0.000 | 0.042 | 0.000 | 0.000 | 0.000 | 0.000 | 0.000 | 0.000 | 0.011 | 0.017 | 0.000 | 0.000 | 0.000 | 0.000 | 0.053 |
|  | **154** | 0.000 | 0.000 | 0.083 | 0.000 | 0.000 | 0.000 | 0.000 | 0.000 | 0.000 | 0.000 | 0.000 | 0.025 | 0.000 | 0.043 | 0.000 | 0.000 | 0.000 | 0.000 | 0.011 | 0.117 |
|  | **155** | 0.000 | 0.000 | 0.000 | 0.045 | 0.000 | 0.000 | 0.000 | 0.000 | 0.000 | 0.000 | 0.000 | 0.000 | 0.031 | 0.000 | 0.000 | 0.000 | 0.000 | 0.229 | 0.000 | 0.000 |
|  | **156** | 0.000 | 0.083 | 0.000 | 0.000 | 0.000 | 0.000 | 0.000 | 0.087 | 0.233 | 0.141 | 0.234 | 0.300 | 0.042 | 0.394 | 0.069 | 0.000 | 0.053 | 0.427 | 0.152 | 0.266 |
|  | **157** | 0.104 | 0.125 | 0.000 | 0.182 | 0.156 | 0.000 | 0.000 | 0.022 | 0.081 | 0.000 | 0.021 | 0.000 | 0.031 | 0.074 | 0.293 | 0.250 | 0.000 | 0.042 | 0.098 | 0.032 |
|  | **158** | 0.000 | 0.042 | 0.000 | 0.000 | 0.000 | 0.184 | 0.552 | 0.043 | 0.000 | 0.027 | 0.000 | 0.000 | 0.229 | 0.128 | 0.034 | 0.000 | 0.170 | 0.052 | 0.163 | 0.096 |
|  | **159** | 0.083 | 0.292 | 0.833 | 0.045 | 0.406 | 0.474 | 0.125 | 0.717 | 0.663 | 0.826 | 0.681 | 0.575 | 0.417 | 0.223 | 0.017 | 0.350 | 0.128 | 0.250 | 0.370 | 0.096 |
|  | **160** | 0.813 | 0.271 | 0.042 | 0.727 | 0.375 | 0.079 | 0.281 | 0.109 | 0.000 | 0.000 | 0.000 | 0.050 | 0.156 | 0.043 | 0.448 | 0.110 | 0.000 | 0.000 | 0.098 | 0.053 |
|  | **161** | 0.000 | 0.000 | 0.000 | 0.000 | 0.000 | 0.211 | 0.000 | 0.000 | 0.000 | 0.005 | 0.000 | 0.050 | 0.010 | 0.000 | 0.000 | 0.290 | 0.596 | 0.000 | 0.076 | 0.021 |
|  | **162** | 0.000 | 0.000 | 0.000 | 0.000 | 0.000 | 0.000 | 0.000 | 0.000 | 0.000 | 0.000 | 0.000 | 0.000 | 0.021 | 0.000 | 0.000 | 0.000 | 0.000 | 0.000 | 0.000 | 0.032 |
|  | **163** | 0.000 | 0.000 | 0.000 | 0.000 | 0.000 | 0.053 | 0.000 | 0.000 | 0.000 | 0.000 | 0.000 | 0.000 | 0.000 | 0.000 | 0.000 | 0.000 | 0.053 | 0.000 | 0.000 | 0.000 |
|  | **164** | 0.000 | 0.000 | 0.000 | 0.000 | 0.000 | 0.000 | 0.000 | 0.000 | 0.000 | 0.000 | 0.000 | 0.000 | 0.000 | 0.000 | 0.000 | 0.000 | 0.000 | 0.000 | 0.000 | 0.011 |
|  | **165** | 0.000 | 0.000 | 0.000 | 0.000 | 0.000 | 0.000 | 0.000 | 0.000 | 0.000 | 0.000 | 0.000 | 0.000 | 0.000 | 0.000 | 0.000 | 0.000 | 0.000 | 0.000 | 0.000 | 0.021 |
| **A9** | **N** | 19 | 21 | 22 | 21 | 13 | 17 | 48 | 23 | 43 | 92 | 47 | 19 | 48 | 47 | 29 | 50 | 47 | 48 | 47 | 47 |
|  | **182** | 0.421 | 0.024 | 0.205 | 0.429 | 0.308 | 0.000 | 0.052 | 0.065 | 0.000 | 0.293 | 0.362 | 0.053 | 0.000 | 0.021 | 0.741 | 0.070 | 0.223 | 0.583 | 0.298 | 0.351 |
|  | **188** | 0.368 | 0.381 | 0.545 | 0.571 | 0.346 | 0.765 | 0.469 | 0.630 | 0.500 | 0.397 | 0.489 | 0.447 | 0.354 | 0.213 | 0.034 | 0.930 | 0.415 | 0.115 | 0.117 | 0.170 |
|  | **189** | 0.000 | 0.000 | 0.000 | 0.000 | 0.000 | 0.000 | 0.000 | 0.000 | 0.000 | 0.000 | 0.000 | 0.000 | 0.000 | 0.000 | 0.000 | 0.000 | 0.000 | 0.094 | 0.000 | 0.000 |
|  | **191** | 0.211 | 0.595 | 0.250 | 0.000 | 0.346 | 0.206 | 0.396 | 0.283 | 0.500 | 0.310 | 0.149 | 0.500 | 0.646 | 0.766 | 0.224 | 0.000 | 0.362 | 0.208 | 0.319 | 0.436 |
|  | **194** | 0.000 | 0.000 | 0.000 | 0.000 | 0.000 | 0.029 | 0.083 | 0.022 | 0.000 | 0.000 | 0.000 | 0.000 | 0.000 | 0.000 | 0.000 | 0.000 | 0.000 | 0.000 | 0.266 | 0.043 |
|  |  |  |  |  |  |  |  |  |  |  |  |  |  |  |  |  |  |  |  |  |  |
|  |  |  |  |  |  |  |  |  |  |  |  |  |  |  |  |  |  |  |  |  |  |
| Na |  | 3.33 | 3.44 | 3.12 | 3.27 | 3.5 | 3.25 | 3.53 | 3.28 | 3.34 | 3.25 | 3.28 | 3.64 | 3.68 | 3.6 | 2.95 | 2.46 | 3.06 | 3.19 | 4.08 | 4.44 |
| Np |  | 0.00 | 0.08 | 0.07 | 0.05 | **0.14** | 0.02 | 0.01 | 0.06 | 0.00 | 0.06 | 0.00 | **0.21** | 0.05 | 0.04 | 0.04 | 0.00 | 0.01 | **0.22** | 0.04 | **0.17** |
| Ho |  | 0.448 | 0.467 | 0.407 | 0.434 | 0.435 | 0.573 | 0.521 | 0.399 | 0.465 | 0.424 | 0.504 | 0.507 | 0.523 | 0.550 | 0.546 | 0.418 | 0.391 | 0.429 | 0.571 | 0.674 |
| He |  | 0.446 | 0.516 | 0.438 | 0.435 | 0.519 | 0.503 | 0.554 | 0.404 | 0.491 | 0.454 | 0.497 | 0.529 | 0.547 | 0.532 | 0.451 | 0.341 | 0.458 | 0.459 | 0.582 | 0.631 |
